# Синдром Коккейна: особенности клинической манифестации и алгоритм наблюдения в детском возрасте

**DOI:** 10.14341/probl13634

**Published:** 2026-03-07

**Authors:** А. Л. Кунгурцева, А. В. Попович, Ю. В. Тихонович, Т. Е. Иванникова, В. А. Ковальская, П. А. Васильев, А. В. Витебская

**Affiliations:** ФГАОУ ВО Первый МГМУ им. И.М. Сеченова Минздрава России (Сеченовский университет)Россия; Sechenov First Moscow State Medical UniversityRussian Federation; Медико-генетический научный центр им. академика Н.П. БочковаРоссия; Research Centre for Medical GeneticsRussian Federation

**Keywords:** синдром Коккейна, синдромы преждевременного старения, репарация ДНК, прогероидные синдромы, Cockayne syndrome, premature aging syndromes, DNA repair, progeroid syndromes

## Abstract

**ОБОСНОВАНИЕ:**

ОБОСНОВАНИЕ. Синдром Коккейна — ультраредкое (1:2,5 млн) наследственное заболевание из группы прогероидных синдромов, обусловленное патогенными и вероятнопатогенными вариантами в генах репарации ДНК (ERCC8, ERCC6, XPB (ERCC3), XPD (ERCC2) и XPG (ERCC5)) и характеризующееся аномальной фоточувствительностью, врожденной катарактой, микроцефалией, нейросенсорной тугоухостью, патологией нервной системы и другими мультисистемными изменениями. В данной рукописи, впервые в Российской Федерации, представлены результаты клинико-генетического исследования и наблюдения за российской когортой пациентов.

**МАТЕРИАЛЫ И МЕТОДЫ:**

МАТЕРИАЛЫ И МЕТОДЫ. В течение 2 лет, с 2023 по 2025 гг., под клиническим наблюдением находились 7 пациентов с синдромом Коккейна (4 девочки и 3 мальчика) в возрасте от 3 лет 11 месяцев до 16 лет 3 месяцев, из них у 3 пациентов был диагностирован синдром Коккейна типа А (причинные варианты в гене ERCC8), у 4 пациентов — тип В (причинные варианты в гене ERCC6). Всем пациентам проводилось комплексное мультидисциплинарное обследование с оценкой результатов лабораторных и инструментальных методов исследования.

**РЕЗУЛЬТАТЫ:**

РЕЗУЛЬТАТЫ. По данным наблюдений, нами подтверждена неполная корреляция между генотипом и фенотипом, описанная ранее в литературе. При генотипе синдрома Коккейна типа В, ранее коррелирующего с тяжелым течением заболевания, только у одного пациента наблюдалось тяжелое течение синдрома, у двух — умеренной степени, у одного пациента — легкое течение, что свидетельствует о вариабельности клинической картины в рамках поражения одного гена, а тяжесть течения коррелировала скорее с возрастом дебюта заболевания: раннее начало (до 1 года) ассоциировалось с более быстрым прогрессированием заболевания. Также, вне зависимости от генотипа и степени тяжести течения заболевания, большие диагностические критерии были выявлены у всех пациентов: врожденная катаракта была диагностирована у 5 из 7 наблюдаемых пациентов, нейросенсорная тугоухость — у двух пациентов умеренного и легкого течения заболевания, прогрессирующая патология нервной системы — у 6 пациентов из 7, микроцефалия была диагностирована у всех пациентов.

**ЗАКЛЮЧЕНИЕ:**

ЗАКЛЮЧЕНИЕ. Проведенное исследование расширяет понимание естественного течения синдрома Коккейна и наши знания о вариабельности клинических проявлений и тяжести течения заболевания в рамках поражения одного гена. Своевременная диагностика и персонализированный подход мультидисциплинарной команды специалистов способны замедлить прогрессирование осложнений и улучшить качество жизни пациентов. Работа представляет ценность для врачей различных специальностей, занимающихся диагностикой и лечением орфанных генетических заболеваний, а также исследователей, изучающих механизмы репарации ДНК и преждевременное старение.

## Abstract (en)

**BACKGROUND:**

BACKGROUND: Cockayne syndrome is an ultra-rare (1:2.5 million) hereditary disease from the group of progeroid syndromes caused by pathogenic and probable-pathogenic variants in DNA repair genes (ERCC8, ERCC6, XPB (ERCC3), XPD (ERCC2) and XPG (ERCC5)) and characterized by abnormal photosensitivity, congenital cataract, microcephaly, sensorineural hearing loss, nervous system pathology and other multisystem changes. In this manuscript, for the first time in the Russian Federation, we present the results of a clinical and genetic study and follow-up of a Russian cohort of patients.

**MATERIALS AND METHODS:**

MATERIALS AND METHODS: During 2 years, from 2023 to 2025, 7 patients with Cockayne syndrome (4 girls and 3 boys) aged from 3 years 11 months to 16 years 3 months were under clinical observation, of whom 3 patients were diagnosed with Cockayne syndrome type A (causative variants in ERCC8 gene) and 4 patients with type B (causative variants in ERCC6 gene). All patients underwent a comprehensive multidisciplinary examination with evaluation of the results of laboratory and instrumental methods of investigation.

**RESULTS:**

RESULTS: Based on observational data, we confirmed the incomplete correlation between genotype and phenotype previously described in the literature. With the genotype of Cockayne syndrome type B, previously correlated with severe course of the disease, only one patient had a severe course of the syndrome, two patients had a moderate course, and one patient had a mild course, indicating the variability of the clinical picture within a single gene lesion, and the severity of the course correlated rather with the age of the disease debut: early onset (before 1 year of age) was associated with faster disease progression. Also, regardless of the genotype and severity of the disease course, major diagnostic criteria were identified in all patients: congenital cataract was diagnosed in 5 of 7 observed patients, sensorineural hearing loss in two patients of moderate and mild course of the disease, progressive pathology of the nervous system in 6 of 7 patients, and microcephaly was diagnosed in all patients.

**CONCLUSION:**

CONCLUSION: This study expands our understanding of the natural course of Cockayne syndrome and our knowledge of the variability of clinical manifestations and severity of the disease course within a single gene lesion. Timely diagnosis and personalized approach of a multidisciplinary team of specialists can slow the progression of complications and improve the quality of life of patients. The work is of value for physicians of various specialties involved in the diagnosis and treatment of orphan genetic diseases, as well as researchers studying the mechanisms of DNA repair and premature aging.

Синдром Коккейна — это ультраорфанное (ультраредкое) наследственное заболевание, относящееся к группе сегментарных прогероидных синдромов, связанных с нарушением репарации ДНК. Заболевание имеет аутосомно-рецессивный тип наследования, обусловлено компаунд-гетерозиготными и гомозиготными патогенными и вероятно-патогенными вариантами в генах ERCC8, ERCC6, XPB (ERCC3), XPD (ERCC2) и XPG (ERCC5). Распространенность заболевания составляет 1 случай на 2,5 млн населения [1–2].

Синдром был впервые описан в 1936 г. Эдвардом Коккейном (Edward Cockayne) как «карликовость с атрофией сетчатки и глухотой» на примере двух сибсов (девочка 7 лет 11 мес и мальчик 6 лет 3 мес) с микроцефалией, низкорослостью и диспропорциональным строением, умственной отсталостью, снижением слуха и зрения [1][3].

С современной точки зрения врожденная катаракта, нейросенсорная тугоухость и микроцефалия с прогрессирующей неврологической патологией являются классическими диагностическими критериями синдрома Коккейна. Врожденная катаракта выявляется у большинства пациентов вне зависимости от тяжести клинического течения заболевания. Прогрессирование микроцефалии и снижения слуха в раннем возрасте свидетельствуют о тяжелом течении синдрома [1][4][5].

Кроме этого клиническая картина характеризуется умеренной задержкой психомоторного развития (ЗПМР), снижением темпов роста и прибавки массы тела, задержкой речевого развития, формированием контрактур больших суставов с последующим регрессом моторных навыков и прогрессирующими неврологическими нарушениями. При тяжелом течении данные клинические проявления отмечаются на 1-м году жизни; при умеренной степени тяжести — после 2 лет; при легком течении — в подростковом возрасте. При тяжелом течении отмечается ранняя инвалидизация и высокая смертность, а средняя продолжительность жизни варьирует от 3 до 5 лет; при умеренной степени тяжести — в среднем 16,1 года, при легкой пациенты доживают до 50 лет и более [1][4].

Для пациентов с патогенными и вероятно-патогенными вариантами в гене ERCC8 (синдром Коккейна, тип А по генетической классификации) чаще характерно течение заболевания умеренной степени тяжести (I тип по клинической классификации), в то время как у пациентов с патогенными и вероятно-патогенными вариантами в гене ERCC6 (синдром Коккейна, тип В по генетической классификации) чаще встречается более тяжелое течение (II тип по клинической классификации), однако не во всех случаях тяжесть течения соответствует генотипу [4].

## Описание клинических случаев

С 2023 по 2025 гг. нами велось наблюдение за 7 неродственными пациентами с синдромом Коккейна (4 девочки и 3 мальчика) в возрасте 3,11–16,3 года. У 3 пациентов был диагностирован синдром Коккейна тип А (2 девочки и 1 мальчик), у 4 других — тип В (2 девочки и 2 мальчика) (табл. 1).

**Table:** Таблица 1. Особенности перинатального периода и клинические характеристики пациентов до постановки диагноза Примечания: *врожденный вертикальный таран — врожденное вертикальное положение таранной кости; ХФПН — хроническая фетоплацентарная недостаточность; ЗПМР — задержка психомоторного развития; ПП ЦНС — перинатальные поражения центральной нервной системы; ПЖК — подкожно-жировая клетчатка; выделенные вероятно-патогенные варианты — ранее не описаны в базе данных Human Gene Mutation Database v.2025.1 (HGMD).

Пациент №, пол	№1, девочка	№2, мальчик	№3, девочка	№4, девочка	№5, девочка	№6, мальчик	№7, мальчик
Генотип	Гомо-/гемизиготный вариант с.457_458dupAC (p.Cys157fs) в гене ERCC8 (NM_000082.4)	Гомозиготный вариант с. 521delA (p.Lys174fs) в гене ERCC8 (NM_000082.4)	Гомозиготная делеция экзонов 1–4 гена ERCC8 (NM_000082.4) (g.60918265-g.60945078)	Компаунд-гетерозиготные варианты с.1821+1G>А и c.2566C>T (p.Gln856Ter) в гене ERCC6 (NM_000124.4)	Компаунд-гетерозиготные варианты c.2203C>T (p.Arg735*) и c.2566C>T (p.Gln856*) в гене ERСС6 (NM_000124.4)	Гомозиготный вариант с.4146delG (p.Gly1382fs) в гене ERСС6 (NM_000124.4)	Компаунд-гетерозиготные варианты c.304C>T (p.Gln102Ter) и c. 2344dup (p.Ser782Phefs) в гене ERCC6 (NM_000124.4)
Тип синдрома Коккейна	А	А	А	В	В	В	В
Тяжесть течения заболевания	умеренная	умеренная	умеренная	тяжелая	умеренная	умеренная	легкая
Пренатальный период	от 1-й беременности, выраженный токсикоз в 1 триместре	от 2-й беременности (1-я беременность — неразвивающаяся), токсикоз в 1-м триместре, угроза выкидыша в 8 недель, обострение хронического гайморита в 19 недель; кольпит у мамы	от 2-й беременности (1-я беременность — ребенок умер в раннем возрасте), угроза прерывания на всем протяжении, пиелонефрит в 16 недель	от 1-й беременности на фоне железодефицитной анемии, отеков во 2-й половине, ХФПН*, хронического пиелонефрита, в 28 недель — пищевое отравление, в 36 недель — обострение хронической герпетической инфекции	от 1-й беременности, на фоне впервые выявленного гипотиреоза у матери, медикаментозно компенсированного	от 1-й беременности (близкородственный брак), без особенностей	от 3-й беременности (близкородственный брак: мама и папа троюродные брат/сестра), без особенностей; 1-я беременность — девочка (здорова), 2-я беременность — мальчик (здоров)
Роды	41–42 нед., самостоятельные	40 нед., самостоятельные	39 нед., самостоятельные	42 нед., путем кесарева сечения (слабость родовой деятельности)	40 нед., самостоятельные	38–39 нед., самостоятельные	41–42 нед., самостоятельные
При рождении: длина (SDS), масса тела (SDS), оценка по Апгар	51 см (+0,62) 3060 г (-0,92) 9/9 баллов	47 см (-2,01) 2700 г (-2,11) 8/8 баллов	51 см (+0,62) 3300 г (-0,34) 7/8 баллов	55 см (+2,87) 3180 г (-0,63) 8/9 баллов	49 см (-0,51) 2450 г (-2,49) -	49 см (-0,92) 3000 г (-1,34) -	51 см (-0,17) 3250 г (-1,04) 8/9 баллов
Клинические проявления при рождении	врожденная двусторонняя катаракта, врожденный вертикальный таран*	мышечная гипотония, сниженные физиологические рефлексы	врожденная левосторонняя катаракта	врожденная двусторонняя катаракта	диспластическая кардиомиопатия, врожденный вертикальный таран*	врожденная двусторонняя катаракта, двусторонний крипторхизм	врожденная двусторонняя катаракта
Клинические проявления до 1 года	с 9 мес. — мышечная дистония, умеренная ЗПМР*	с 1‑го мес. жизни — ПП ЦНС*, синдром двигательных нарушений, ЗПМР*	с 1‑го мес. жизни — прогрессирующее снижение темпов роста и набора массы тела, умеренная ЗПМР*	с 1‑го мес. — ПП ЦНС*, травматически -гипоксического генеза, ЗПМР*, микроцефалия, сходящееся косоглазие с 5 мес — выраженный регресс моторного развития с 8 мес — нистагм	с 1‑го мес. жизни — ПП ЦНС* гипоксического генеза, ЗПМР*, пилороспазм, двусторонняя дисплазия тазобедренных суставов	-	-
Клинические проявления на момент постановки диагноза	ЗПМР*, задержка роста и дефицит массы тела, синдром мышечной гипотонии (с 2 лет); регресс моторных навыков (с 4 лет); мозжечковая атаксия, контрактуры больших суставов (с 5 лет)	ЗПМР*, задержка речевого развития, мышечная гипотония, дефицит массы тела (с 2 лет); спастический тетрапарез, дизартрия, дефицит когнитивных функций, синдром Фара; дисплазия тазобедренных суставов, плоско-вальгусные стопы 3 степени (с 5 лет);	ЗПМР*, задержка роста, дефицит массы тела (с 4–5 лет); контрактуры больших суставов; дисплазия тазобедренных суставов (с 9–10 лет)	тяжелая ЗПМР*, низкие темпы роста, недостаточное развитие ПЖК* , плоско-вальгусные стопы (со 2‑го года жизни)	ЗПМР*, контрактуры больших суставов, задержка роста и дефицит массы тела, задержка речевого развития (с 4 лет)	ЗПМР*, задержка роста и дефицит массы тела, спастический парапарез, конусовидные конечные фаланги пальцев рук (возраст манифестации неизвестен)	микроцефалия, задержка роста и дефицит массы тела (с 2 лет); легкая задержка психо-речевого развития, ДЦП, смешанная форма (с 6 лет)
Возраст на момент постановки диагноза	6 лет 9 мес	5 лет 6 мес	10 лет 3 мес	2 года 9 мес	5 лет 9 мес	8 лет	9 лет

Из анамнеза известно, что матери 2 пациентов имели отягощенный акушерский анамнез — замершую предыдущую беременность (пациент №2) и смерть ребенка в неонатальном периоде (пациент №3); двое детей были рождены от близкородственного брака (пациенты №6 и №7). Несмотря на различную соматическую патологию, наблюдавшуюся у матерей пациентов во время беременности, ни у одного ребенка не было выявлено каких-либо особенностей развития плода в пренатальный период; результаты неонатального скрининга были в пределах нормальных значений (табл. 1).

Все пациенты родились в результате доношенных беременностей. Показатели длины тела колебались от низких до высоких значений: 51 (47–55) см (медиана (min-max)). Масса тела у всех пациентов была ниже среднепопуляционной: 3060 (2450–3300) г. У 5 пациентов (2 пациентов с типом А, 3 пациентов с типом В) при рождении была диагностирована врожденная катаракта (табл. 1).

6 пациентам (№1–6) диагноз был установлен по результатам проведенного полноэкзомного секвенирования, который был назначен врачом-генетиком в связи с особенностями фенотипа, задержкой психомоторного и физического развития. Пациенту №7, рожденному от близкородственного брака, секвенирование клинического экзома было проведено в связи с семейным анамнезом пигментной ксеродермой, выявленной у двоюродного брата; у родителей пробанда выполнено секвенирование по Сэнгеру, в результате которого подтверждено бессимптомное носительство патогенных вариантов в гетерозиготном состоянии в гене ERCC6. Средний возраст постановки диагноза — 6,9 (2,9–10,3) года.

Согласно международной базе данных Human Gene Mutation Database v.2025.1 (HGMD), только у пациентов №5 и №7 идентифицированы ранее описанные патогенные варианты (c.2203C>T (p.Arg735*) и c.304C>T (p.Gln102Ter) в гене ERCC6 соответственно), у остальных обследованных пациентов обнаружены ранее не описанные генетические варианты, требующие дальнейшего функционального анализа для подтверждения их патогенности.

Какие-либо фенотипические особенности, ассоциированные с синдромом Коккейна, в раннем неонатальном периоде у детей отсутствовали. Характерный фенотип сформировался у всех пациентов после 2 лет жизни и был представлен выраженным широким лбом, глубоко посажеными глазами, гипоплазией средней и нижней трети лица, микрогнатией, острым подбородком, седловидным носом (рис. 1, 2, 3, 4, 6); килевидной деформацией грудной клетки, недостаточным развитием подкожно-жировой клетчатки (ПЖК) (рис. 5).

**Figure:**
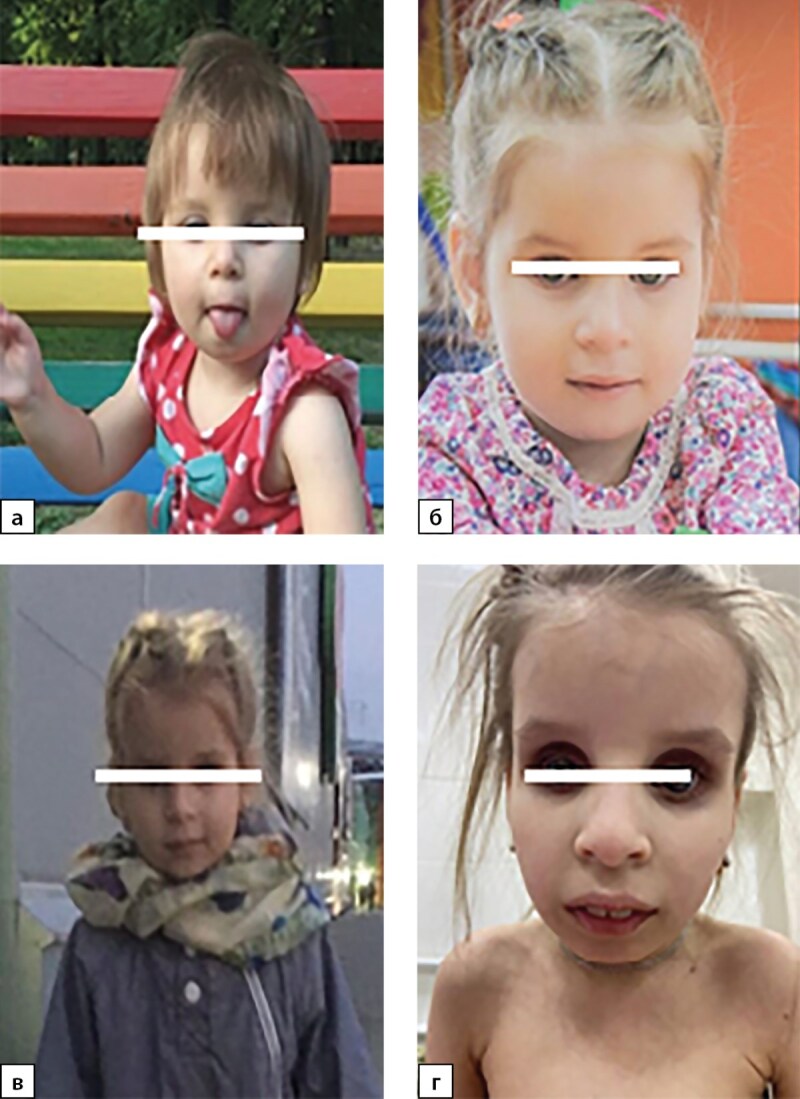
Рисунок 1. Формирование фенотипа, характерного для синдрома Коккейна, у пациентки №1 в первые годы жизни:а) 1 год; б) 2 года; в) 3 года 6 мес; г) 8 лет 3 мес.

**Figure:**
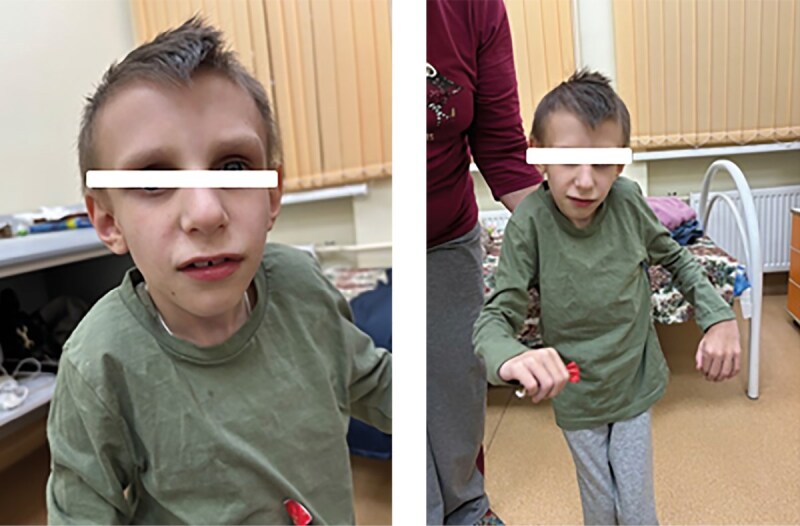
Рисунок 2. Фенотип, характерный для синдрома Коккейна, у пациента №2 в 9 лет 5 мес.

**Figure:**
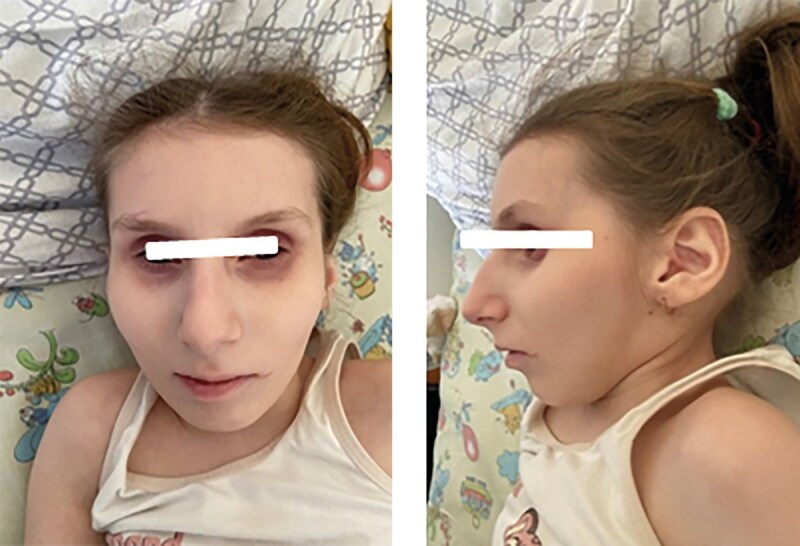
Рисунок 3. Фенотип, характерный для синдрома Коккейна, у пациентки №3 в 13 лет 2 мес.

**Figure:**
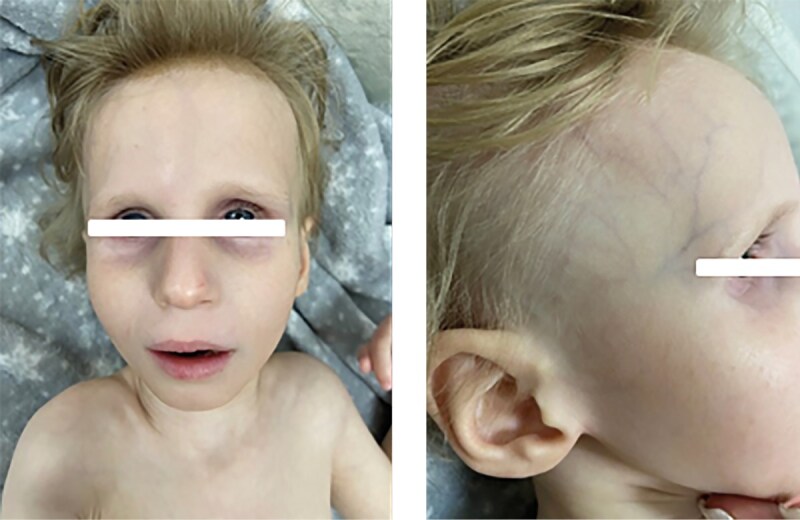
Рисунок 4. Фенотип, характерный для синдрома Коккейна, у пациентки №4 в 3 года 11 мес.

**Figure:**
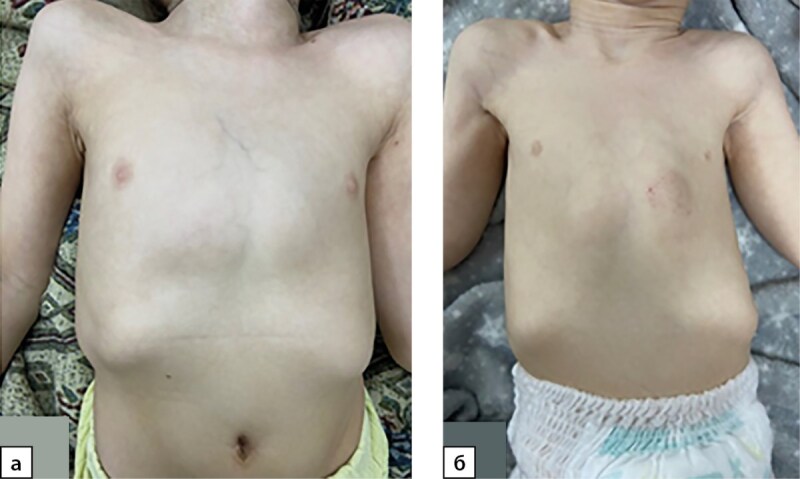
Рисунок 5. Килевидная грудная клетка: а) пациент №1; б) пациент №4

**Figure:**
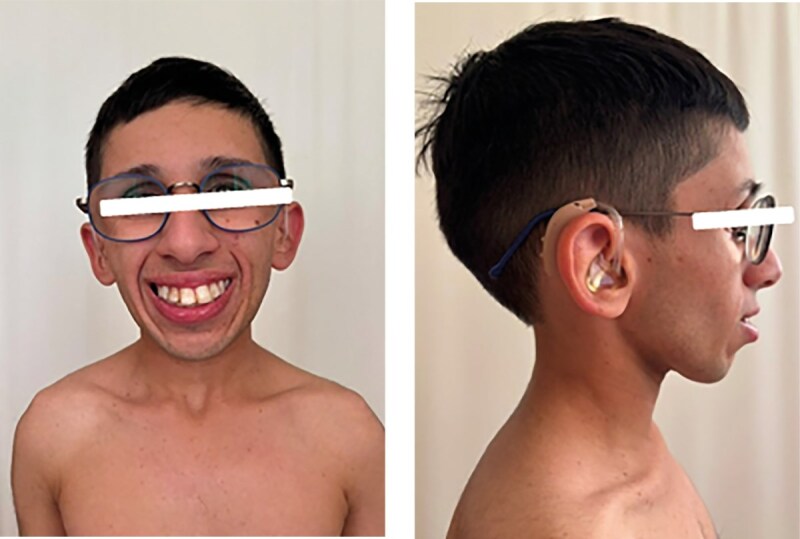
Рисунок 6. Фенотип, характерный для синдрома Коккейна, у пациента №7 в 16 лет 3 мес.

Тяжелое течение синдрома наблюдалось у пациентки №4 (тип В), характеризуясь выраженным регрессом моторного развития с 5‑го месяца жизни, появлением нистагма — с 8‑го месяца жизни. У 4-х пациентов (№1, 2, 3, 5) течение заболевания характеризовалось умеренной степенью тяжести. С 4–5 лет (у пациента №6 — с 7–8 лет) отмечался регресс моторных навыков с последующим развитием неврологической симптоматики, ортопедической патологии когнитивного дефицита, снижением темпов роста и набора массы тела (табл. 1). У пациента №6 наблюдается умеренно-легкое течение заболевания, у пациента №7 (рис. 6) — легкое течение, что свидетельствует о вариабельности клинических проявлений в рамках одного генотипа.

Патологические изменения органов зрения, слуха, нервной, сердечно-сосудистой, пищеварительной, мочевыделительной, эндокринной систем и опорно-двигательного аппарата суммированы в таблице 2.

**Table:** Таблица 2. Мультисистемные изменения у наблюдаемых пациентов Примечание: АЛТ — аланинаминотрансфераза; АСТ —аспартатаминотрансфераза; НЖБП — неалкогольная жировая болезнь печени; ИМТ — индекс массы тела; SDS — Standard Deviation Score; ИФР-1 — инсулиноподобный фактор роста-1; ЛГ — лютеинизирующий гормон; ФСГ — фолликулостимулирующий гормон; МР — магнитно-резонансные; OU — oculus uterque (оба глаза); OD — oculus dexter (правый глаз); OS — oculus sinister (левый глаз); ИОЛ — интраокулярная линза.

Пациент №, пол	№1, девочка	№2, мальчик	№3, девочка	№4, девочка	№5, девочка	№6, мальчик	№7, мальчик
Возраст на момент наиболее позднего обследования	8 лет 2 мес	9 лет 5 мес	13 лет 2 мес	3 года 11 мес	8 лет 8 мес	16 лет 2 мес	16 лет 3 мес
Антропометрические показатели	Рост 98 см (SDS -4,84) Вес 11,3 кг (SDS ИМТ -3,31)	Рост 102,5 см (SDS -5,13) Вес 13 кг (SDS ИМТ -3,28)	Рост 98 см (SDS -8,63) Вес 10 кг (SDS ИМТ -6,88)	Рост 81 см (SDS -4,40) Вес 6,8 кг (SDS ИМТ -6,60)	Рост 110 см (SDS -3,16) Вес 14 кг (SDS ИМТ -3,60)	Рост 103 см (SDS -10,09) Вес 13 кг (SDS ИМТ -6,27)	Рост 116 см (SDS -8,23) Вес 21,6 кг (SDS ИМТ -2,26)
Окружность головы	48 см	-	-	-	43,5 см	46,5 см	49 см
Патология зрения	врожденная двусторонняя катаракта (не оперирована), дистрофия роговицы, косоглазие вторичное постоянное содружественное расходящееся, гиперметропия высокая	гиперметропия средней степени обоих глаз, частичная атрофия зрительных нервов обоих глаз, вторичная левосторонняя экзотропия	врожденная левосторонняя катаракта (состояние после замены ИОЛ), OU — частичная атрофия зрительных путей, хронический кератит, косоглазие вторичное расходящееся, смешанная дистрофия сетчатки, OD — гиперметропия высокая, OS — гиперметропический астигматизм	врожденная двусторонняя катаракта (не оперирована), OU — частичная атрофия зрительного нерва, косоглазие содружественное сходящееся, хронический конъюнктивит	OU — частичная атрофия зрительных нервов, содружественное расходящееся косоглазие, гиперметропия, помутнение роговицы	врожденная двусторонняя катаракта (состояние после замены ИОЛ), помутнение роговицы, расходящееся содружественное косоглазие, нистагм, афакия, двусторонняя атрофия зрительных нервов, гиперметропия	ОU — врожденная катаракта, артифакия, расходящееся косоглазие, ЧАЗН?
Патология слуха	-	-	-	-	-	двусторонняя нейросенсорная тугоухость 3 степени	двусторонняя нейросенсорная тугоухость 3 степени (преимущественно, высокочастотная)
Патология нервной системы	тетрапарез, 2-сторонняя пирамидная недостаточность, мозжечковая атаксия, аномалия Денди-Уокера, тривентрикуляная неоклюзионная гидроцефалия, лейкопатия, гипоплазия миндалин и частично червя мозжечка, дистопия намета мозжечка кверху, атаксия, полинейропатия, когнитивный дефицит	мозжечковая атаксия, аномалия Денди-Уокера, атаксия, когнитивный дефицит	тетрапарез, полиневропатия, МР-картина субтотальной атрофии вещества больших полушарий головного мозга, мозжечка и ствола с признаками вторичного расширения ликворных пространств, перивентрикулярной лейкомаляции; гипо-/аплазии мозолистого тела; неполной инверсии гиппокампов	тетрапарез, тяжелая задержка психического и речевого развития, нистагм	смешанный тетрапарез, атаксический синдром, смешанный экстрапирамидный синдром в форме гипертонически-гипокинетического синдрома, когнитивный дефицит, МР-картина диффузного поражения белого вещества мозга	спастическая дисплегия, выраженная задержка интеллектуального развития, нарушение экспрессивной речи, МР-признаки умеренно выраженного расширения наружных ликворных пространств лобной, теменной областей, признаки ассиметричной внутренней гидроцефалии, кальцинаты вещества мозга правой и левой височной долей	когнитивный дефицит (умственная отсталость)?; полинейропатия?; МР-признаки церебральной и церебеллярной атрофии, с викарным расширением внутренних и наружных ликворных пространств; минимальная гипоплазия мозолистого тела; косвенные признаки кальцификации базальных ядер с обеих сторон
Патология сердечно-сосудистой системы	синусовая тахикардия	синусовая тахикардия, укорочение атриовентрикулярной проводимости, ранняя реполяризация желудочков	наджелудочкая экстрасистолия, предсердный ритм	укорочение атриовентрикулярной проводимости	-	-	выраженная аритмия
Патология опорно-двигательного аппарата	эквино-варусная неврогенная деформация обеих стоп (состояние после операции), дисплазия тазобедренного сустава, контрактуры больших суставов	множественные контрактуры, плоско-вальгусная деформация стоп	сгибательная контрактура коленных суставов, плоско-вальгусная деформация стоп	сгибательные контрактуры 3–4‑го пальцев кистей и лучезапястных суставов, приводящие контрактуры тазобедренных суставов плоско-вальгусная деформация стоп	множественные контрактуры, эквино-варусная неврогенная деформация обеих стоп (состояние после операции), грудопоясничный кифоз	множественные контрактуры, плоско-вальгусная деформация стоп	незначительные контрактуры больших суставов, плоско-вальгусная деформация стоп
Эндокринная патология; стадия полового созревания; костный возраст (по атласу Greulich — Pyle); показатели гормонов	тяжелая задержка роста, дефицит массы тела; половое развитие по Таннеру 1; костный возраст — 6 лет	тяжелая задержка роста, дефицит массы тела; половое развитие по Таннеру 2; костный возраст — 12 лет; ЛГ 11,6 мМЕ/мл, ФСГ 35 мМЕ/мл, тестостерон 14,6 нмоль/л, ИФР-1 286 нг/мл (SDS ИФР-1 +1,9)	тяжелая задержка роста, дефицит массы тела; половое развитие по Таннеру 1–2 (В2 Р1); костный возраст — 10 лет; ЛГ 0,9 мМЕ/мл, ФСГ 6,3 мМЕ/мл, эстрадиол 74 пмоль/л	тяжелая задержка роста, дефицит массы тела; половое развитие по Таннеру 1; данных по костному возрасту нет	тяжелая задержка роста, дефицит массы тела; половое развитие по Таннеру 3; данных по костному возрасту нет	тяжелая задержка роста, дефицит массы тела; данных по половому созреванию нет; данных по костному возрасту нет	тяжелая задержка роста, дефицит массы тела; половое развитие Таннеру 5 (P5;G5); костный возраст — 16,5 года (зоны роста закрыты); ЛГ 5,5 мМЕ/мл; ФСГ 5,5 мМЕ/мл; тестостерон 14,1 нмоль/л; ИФР-1 429 нг/мл (SDS ИФР-1 +0,51)
Патология пищеварительной системы	увеличение размеров печени; повышение трансаминаз (АЛТ 32 ед/л, АСТ 30 ед/л), дисфункция билиарного тракта, синдром дисхолии	энтеропатия неуточненная, дисфункция кардиального сфинктера, дисфункция билиарного тракта; повышение трансаминаз (АЛТ 56 ед/л, АСТ 32 ед/л)	функциональная диспепсия, ГЭРБ, функциональные нарушения билиарного тракта; НЖБП, стеатогепатит (АЛТ 64 ед/л, АСТ 32 ед/л)	увеличение размеров печени; повышение трансаминаз (АЛТ 123 ед/л, АСТ 85 ед/л)	Нет данных	НЖБП, фиброз печени (АЛТ 160 ед/л, АСТ 50,3 ед/л)	хронический(?) гепатит, повышение трансаминаз (АЛТ 136 ед/л, АСТ 44 ед/л); дисфункция биллиарного тракта
Патология почек	-	-	-	-	Хронический пиелонефрит	Правосторонняя пиелоэктазия	-

Нарушение зрения отмечается у всех пациентов (таблица 2). Несмотря на своевременное выявление врожденной двусторонней катаракты, пациентам №1 и №4 оперативные вмешательства не проводились (отказ родителей). Пациенту №7 оперативное вмешательство по замене интраокулярной линзы (ИОЛ) было проведено в возрасте 6 месяцев, пациентам №3 и №6 — в возрасте 4–5 лет. У пациентов №2 и №5 катаракта не диагностирована.

После 2 лет у 6 пациентов (№1–6) была диагностирована частичная атрофия зрительных нервов обоих глаз, дистрофия роговицы, косоглазие вторичное расходящееся, высокая гиперметропия одного или обоих глаз; у пациента №7 наблюдается только расходящееся косоглазие, ЧАЗН(?) с 16,3 года.

В настоящее время всем пациентам проводится очковая коррекция.

Нарушение слуха на сегодняшний день выявлено только у 2 пациентов старшего возраста (№6 и №7) (табл. 2).

Известно, что пациенту №6 возрасте 14 лет, по данным акустической импедансометрии, была диагностирована двусторонняя нейросенсорная тугоухость 3-й степени (пороги визуальной детекции на фоне стимуляции на уровне 60 дБ nHL с двух сторон). Пациенту №7 данный диагноз был установлен в 12 лет. В настоящее время пациентам проводится коррекция слуховым аппаратом.

Остальным 5 пациентам акустическая импедансометрия и/или аудиометрия не проводились.

Неврологические нарушения выявлены у всех пациентов (табл. 2). Только у 1 ребенка с тяжелым течением синдрома (№ 4) микроцефалия отмечалась с 1‑го месяца жизни, у остальных — после 2 лет. В настоящее время окружность головы варьирует с 43,5 см в 8,8 года до 49 см в 16,3 года, что соответствует -2,5 SD.

ЗПМР с первых месяцев жизни была диагностирована у 4 пациентов (№2, 3, 4, 5); у одного ребенка (№1) диагноз установлен в 9 месяцев; пациент №7 до 2 лет развивался без особенностей; данные по раннему развитию пациента №6 отсутствуют.

Возраст, когда был отмечен выраженный регресс развития детей варьировал от 2 лет (№2) до 5 (№1, 3, 4, 5, 6) или 6 лет (№7). У половины пациентов (№ 1, 3, 5) нарастание тяжести клинических проявлений отмечено после перенесенной респираторной инфекции.

Прогрессирующая ЗПМР сопровождалась проявлениями мозжечковой атаксии, отсутствием прогрессии речевого развития; у 1 ребенка (№5) также выявлен смешанный экстрапирамидный синдром в форме гипертонически-гипокинетического синдрома.

По данным МРТ головного мозга, у всех пациентов были выявлены МР-признаки, характерные для синдрома Коккейна: аномалии Денди-Уокера, признаки синдрома Фара (кальцификация базальных ганглиев), субтотальная атрофия вещества больших полушарий головного мозга, мозжечка и ствола с признаками вторичного расширения ликворных пространств, перивентрикулярной лейкомаляции; гипо-/аплазии мозолистого тела; у 2 пациентов (№1 и №6) — МР-признаки неокклюзионной гидроцефалии.

В настоящее время у одного пациента (№7) сохраняется возможность самостоятельной ходьбы и полная возможность обслуживать себя в быту, у одного пациента (№2) — ходьба с опорой (рис. 4), у остальных 5 пациентов диагностирован тетрапарез.

У всех пациентов отмечается когнитивный дефицит.

Сердечно-сосудистые нарушения у наблюдаемых пациентов были представлены неспецифическими изменениями, выявленными при проведении ЭКГ (табл. 2).

По результатам эхокардиографии (ЭхоКГ), ультразвуковой допплерографии (УЗДГ) брахиоцефальных сосудов данных за патологические изменения получено не было; липидный профиль у всех пациентов — без особенностей.

Патология опорно-двигательного аппарата представлена врожденными деформациями стопы и приобретенными контрактурами суставов (табл. 2). У 2 пациентов (№1, 5) на 1-м году жизни был диагностирован врожденный вертикальный таран (ВВТ), характеризующийся плоско-вальгусной деформацией стопы тяжелой степени. Данным пациентам проводилась хирургическая коррекция деформаций в возрасте 7 лет (рис. 7). У 4 пациентов (№2, 3, 4, 6) диагностированы плоско-вальгусные деформации стоп умеренной степени.

**Figure:**
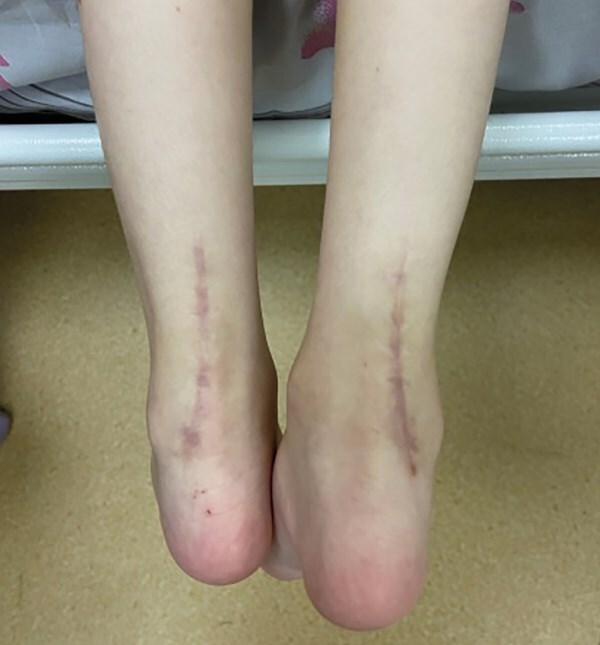
Рисунок 7. Врожденный вертикальный таран (состояние после ахиллопластики).

За время наблюдения также были выявлены сгибательные контрактуры коленных суставов, контрактуры малых суставов (у всех пациентов), у 1 пациента (№5) — кифосколиоз.

Эндокринная патология представлена тяжелой задержкой роста, которая наблюдается у всех пациентов (SDS роста варьирует от -3,16 до -10,09), и дефицитом массы тела (SDS ИМТ от -2,26 до -6,88). Низкие темпы набора веса отмечались у детей с рождения, снижение темпов роста стало заметно после 1,5–2 лет. Уровень инсулиноподобного фактора роста-1 (ИФР-1) у всех пациентов соответствует возрасту и стадии полового развития; нарушения функции щитовидной железы и надпочечников не отмечалось (табл. 2).

У 4 пациентов пубертатного возраста (№2, 3, 5,7) отмечена самостоятельная инициация пубертата. Сроки начала полового созревания варьировали от ранних (пациентка №5 — 7,5 года, пациент №2 — 9 лет, пациент №7 — 8 лет) до поздних (пациентка №3 — 13 лет). У пациента №2 с учетом высокого уровня ФСГ нельзя исключить начало формирования гипергонадотропного гипогонадизма (табл. 2).

У 3 пациентов допубертатного возраста костный возраст, по данным рентгенографии кистей рук, в среднем отстает от паспортного на 2 года.

Патология пищеварительной системы характеризуется умеренным повышением уровня печеночных ферментов (уровень аланинаминотрансферазы (АЛТ) варьирует от 32 ед/л до 160 ед/л; уровень аспартатаминотрансферы (АСТ) — от 30 ед/л до 85 ед/л). Все пациенты находятся на терапии урсодезоксихолевой кислотой (УДХК).

У 3 пациентов (№1, 3, 4) на УЗИ органов брюшной полости регистрируются УЗ-признаки увеличения печени, усиление сосудистого рисунка печени; у 1 пациента (№6) — УЗ-признаки фиброза печени.

Нарушение функции почек у наблюдаемых нами пациентов не выявлено. У одного мальчика (№6) диагностирована правосторонняя пиелоэктазия, у одной девочки (№5) — хронический пиелонефрит.

## Обсуждение

Синдром Коккейна — ультраорфанное наследственное заболевание, которое возникает в результате патогенных вариантов в генах ERCC8 (5q12-q31), ERCC6 (10q11), XPB (ERCC3), XPD (ERCC2) и XPG (ERCC5) [1][2][6][7]. Эти гены кодируют белки, участвующие в эксцизионной репарации нуклеотидов (nucleotide excision repair (NER)), что является одним из универсальных механизмов репарации ДНК. Патогенные и вероятно-патогенные варианты вызывают нарушение, ассоциированное с повреждением транскрипционно активной ДНК ультрафиолетовым (УФ) излучением. УФ-индуцированное повреждение ДНК приводит к замедлению репарации ДНК, преждевременному апоптозу клеток и, как следствие, к преждевременному старению [8–13].

Особенностью синдрома Коккейна является отсутствие высоких рисков злокачественных новообразований (ЗНО), в отличие от других синдромов, связанных с нарушением репарации ДНК. Предполагается, что это связано с ингибированием клеточного роста из-за повышенного апоптоза клеток с поврежденной ДНК, однако в настоящее время данный вопрос остается предметом дискуссий [14].

Несмотря на то, что заболевание относится к группе синдромов преждевременного старения, доказано, что длина теломер и/или активность теломеразы у пациентов с синдромом Коккейна не отличается от таковой у здоровых детей того же возраста [1].

В зависимости от генотипа выделяют четыре типа синдрома Коккейна: патогенные и вероятно-патогенные варианты в гене ERCC8 (5q12-q31, OMIM 216400) ассоциированы с развитием синдрома Коккейна типа А, в гене ERCC6 (10q11, OMIM 133540) — типа В, в генах XPB (ERCC3, 2q14.3, OMIM 610651), XPD (ERCC2, 19q13.32, OMIM 278730) и XPG (ERCC5, 13q33.1, OMIM 278780) — с редким сочетанием синдрома Коккейна с пигментной ксеродермой, а также описаны для синдрома чувствительности к УФ-излучению (англ. ultraviolet sensitivity syndrome, UVSS), который, по мнению разных авторов, также считается частью спектра синдрома Коккейна [1][6][7].

В связи с патогенезом заболевания, ассоциированным с аномальной фоточувствительностью, пациентам следует придерживаться техники безопасности при нахождении рядом с некоторыми видами искусственного освещения (неоновым и галогеновым) и на улице в дневное время: использовать на все участки кожи крема с SPF-50 каждые 2 часа нахождения на улице и солнцезащитные очки с целью предупреждения развития ожога роговицы [2].

Клинически заболевание подразделяется на 4 типа течения (табл. 3) [1][4].

**Table:** Таблица 3. Типы течения синдрома Коккейна [1][4][15–21] Примечание: ЗНО — злокачественное новообразование.

Клинический тип	I тип (классический)	II тип	III тип	Сочетание синдрома Коккейна и пигментной ксеродермы
	Церебро-окуло-фацио-скелетный синдром (Cerebro-oculo-facio-skeletal syndrome (COFS)	корреляция «генотип-фенотип»
XPB (ERCC3)	XPD (ERCC2)	XPG (ERCC5)
Возраст манифестации	1–2 год жизни	с первых месяцев жизни до 1 года жизни	пренатальный период	после 2-х лет, часто — в подростковом возрасте	после 2-х лет	пренатальный период	пренатальный период
Тяжесть течения	умеренная	тяжелая	крайне тяжелая	легкая	легкая	тяжелая	тяжелая
Клинические проявления	регресс моторных навыков, специфический фенотип; классические проявления синдрома	пре - и постнатальная задержка роста, врожденная микроцефалия, ранние послеродовые контрактуры позвоночника и суставов	внутриутробная задержка роста, пренатальная микроцефалия, артрогрипоз	задержка роста с легкими неврологическими нарушениями, патологией зрения; нормальное речевое развитие и отсутствие снижения интеллекта	нормальный рост и вес, гипогонадизм, поздняя невропатия со смешанными демиелинизирующими и аксональными признаками, иногда — развитие рака кожи	внутриутробная задержка роста, пренатальная микроцефалия, крипторхизм, врожденная катаракта, пигментные изменения и атрофия кожи, множественные ЗНО кожи	клинические проявления схожи с генотипом XPD (ERCC2), однако ЗНО кожи не распространено
Средняя продолжительность жизни	16,1 года	5 лет	до 3-х лет	неизвестна	до 50 лет и более	неизвестна	неизвестна

По данным литературы, патогенные и вероятно-патогенные варианты в гене ERCC8 (синдром Коккейна типа А) чаще ассоциированы с умеренно тяжелым клиническим течением (клинический тип I), а в гене ERCC6 (синдром Коккейна типа В) — с более тяжелым клиническим течением типа II [4]. Среди наблюдаемых нами пациентов тяжелое течение, которое может быть отнесено к типу II, наблюдалось у пациента №4 с синдромом Коккейна типа В; у двух других пациентов (№5 и №6) с типом В течение заболевания не отличалось от того, что мы наблюдали у пациентов с синдромом Коккейна типа А; у пациента №7 с типом В наблюдалось легкое течение III типа, однако проведение четкой корреляции генотип-фенотип затруднительно в связи с малой выборкой пациентов (табл. 1).

Независимо от клинического типа, заболевание характеризуется множественными мультисистемными изменениями. Большими (классическими) проявлениями синдрома Коккейна являются врожденная катаракта, нейросенсорная тугоухость и микроцефалия с прогрессирующей неврологической патологией, которые выявляются у большинства пациентов [1][4]. Врожденная катаракта была диагностирована у 5 из 7 наблюдаемых нами пациентов независимо от генотипа, что согласуется с литературными данными.

Считается, что возраст манифестации снижения слуха напрямую коррелирует с тяжестью течения синдрома Коккейна [1][4][5]. Среди наблюдаемых нами пациентов нейросенсорная тугоухость диагностирована только у двух детей (№6 и №7) в возрасте 14 и 12 лет соответственно, что подтверждает умеренно-легкую степень тяжести течения заболевания. Остальные пациенты значительно младше, поэтому нарушение слуха могло еще не успеть сформироваться.

Микроцефалия — важнейший критерий синдрома Коккейна, а ее ранняя манифестация ассоциирована с тяжелым течением заболевания в дальнейшем [1][4]. Так, у 6 пациентов (№1, 2, 3, 5, 6, 7) прогрессирующая микроцефалия отмечалась с 2 лет, и в дальнейшем у 5 пациентов (№1, 2, 3, 5, 6) сохранялась умеренная степень тяжести течения заболевания, у пациента №7 — легкое течение, тогда как у пациента №4 отсутствие увеличение объема головы наблюдалось с 1‑го месяца жизни, и в дальнейшем отмечено тяжелое течение заболевания.

Прогрессирующая патология нервной системы является ключевым проявлением синдрома Коккейна и определяет тяжесть течения и степень инвалидизации. Зачастую в связи со схожестью проявлений, в первые годы жизни пациентам ошибочно устанавливается диагноз детский церебральный паралич [3][4].

Такие проявления, как ЗПМР, регресс моторных навыков, прогрессирующая атрофия головного мозга, проявления периферической нейропатии, судорожные расстройства и тремор, МР-признаки атрофии вещества головного мозга, двусторонняя кальцификация базальных ганглиев, зубчатого ядра и подкоркового белого вещества проявляются у большинства пациентов при умеренной и тяжелой степени тяжести течения синдрома Коккейна. В связи с прогрессирующей патологией нервной системы пациентам рекомендовано избегать приема опиоидов и седативных средств в связи с повышенной чувствительностью к данным препаратам [1][2][22][23].

Патология опорно-двигательного аппарата представлена формированием грудопоясничного кифоза, приобретенными контрактурами суставов и врожденными деформациями стопы, наиболее распространенные из которых, плоско-вальгусные деформации, в том числе их тяжелые врожденные формы — ВВТ [24][25]. Так, у 2 пациентов (№1 и №5) с ВВТ была проведена ахиллопластика с целью коррекции тяжелой деформации стопы, остальным пациентам (№2, 3, 4, 6, 7) с умеренной степенью деформаций стопы коррекция проводится неинвазивными методами (ношение ортопедической обуви, ортопедических стелек).

Для пациентов с синдромом Коккейна характерно раннее развитие сердечно-сосудистой патологии: атеросклероза, кардиомиопатии, артериальной гипертензии, фиброзных и кальцифицирующих васкулитов, редко — инсульта. Перечисленные изменения развиваются в более старшем возрасте, чаще — во втором десятилетии жизни [26–28], что объясняет их отсутствие у наших пациентов.

Согласно нашим наблюдениям, как и по данным литературы, патология эндокринной системы при синдроме Коккейна преимущественно представлена низкими темпами роста и набора массы тела в среднем после 1‑го года жизни с последующей прогрессирующей задержкой роста и дефицитом массы тела. На сегодняшний день в литературе нет описаний применения рекомбинантного гормона роста у пациентов с синдромом Коккейна, однако его использование не рекомендовано, что, вероятнее всего, связано с патогенезом заболевания [29].

Дефицит массы тела, как правило, обусловлен недостаточным развитием подкожно-жировой клетчатки и сниженным аппетитом, в связи с чем всем пациентам рекомендован прием высокобелковых смесей дополнительно к основным приемам пищи, а прогрессирующая неврологическая патология может потребовать помощь в кормлении, в том числе с использованием назогастрального зонда или гастростомы [2].

Патологические изменения секреции гормонов щитовидной железы, надпочечников, нарушения углеводного и липидного обменов не характерны. В 30% случаев у мужчин с синдромом Коккейна развивается гипогонадизм, у женщин — нерегулярные менструации. Однако описан случай рождения ребенка пациенткой с синдромом Коккейна [2][27].

Патология пищеварительной системы при синдроме Коккейна представлена поражением печени в виде умеренного повышения уровня печеночных ферментов, развития печеночно-клеточной недостаточности, холестатического поражения печени [30][31]. По результатам наших наблюдений, у всех пациентов с раннего возраста отмечается умеренное повышение уровня печеночных ферментов (АЛТ, АСТ), что необходимо учитывать при динамическом обследовании пациентов с данной патологией. Всем пациентам была рекомендована терапия УДХК.

Также в настоящее время в связи с описанием серии случаев развития острой печеночной недостаточности у данной группы пациентов, в том числе с летальным исходом, синдром Коккейна рассматривается как абсолютное противопоказание к применению метронидазола (противопротозойный и противомикробный лекарственный препарат). При необходимости применения и отсутствия альтернативного лечения необходимо контролировать функциональные показатели печени и состояние свертывающей системы крови в течение 2–4 недель [32][33].

По данным литературы, для 60–70% пациентов с синдромом Коккейна характерна патология почек — микроальбуминурия, гиперальдостеронизм без почечной недостаточности или стеноза почечных артерий, сегментарный гломерулосклероз, артериосклероз, тубулоинтерстициальный фиброз или канальцевая атрофия [34][35]. Отсутствие тяжелой почечной патологии у наших пациентов, вероятно, объясняется ранним возрастом наблюдаемых.

В настоящее время патогенетического лечения синдрома Коккейна не разработано, однако предпринимаются попытки создания генно-терапевтических препаратов. В 2020 г. S.Wang и соавт. опубликовали результаты генного редактирования индуцированных плюрипотентных стволовых клеток из фибробластов пациента с синдромом Коккейна с гетерозиготными мутациями в гене ERCC6 (c.643G>T в экзоне 4 и c.3776C>A в экзоне 18) методом CRISPR/Cas9. Полученные мезенхимальные стволовые клетки демонстрировали высокий жизненный потенциал, сохраняли высокую стабильность генома и не образовывали опухолей in vivo. В настоящее время исследования продолжаются [36].

## Заключение

Учитывая мультисистемный характер поражения и неуклонно прогрессирующее течение заболевания, пациентам с синдромом Коккейна необходимо регулярное наблюдение мультидисциплинарной команды врачей, в которую должны входить специалисты по всем основным составляющим синдрома: педиатр, генетик, офтальмолог, оториноларинголог, сурдолог, невролог, детский кардиолог, детский эндокринолог, травматолог-ортопед.

С учетом описанной патологии пациентам с синдромом Коккейна необходимо ежегодное обследование [1][4][15][22][29][30]:

- молекулярно-генетическое исследование (полноэкзомное/полногеномное секвенирование) — однократно, при постановке диагноза;

- контроль антропометрических показателей (рост, вес);

- общеклиническое исследование крови;

- биохимическое исследование крови (общий белок, АСТ, АЛТ, свободный и связанный билирубин, креатинин, глюкоза);

- ЭКГ;

- МРТ головного мозга;

- аудиометрия;

-рентгенография позвоночника, больших и малых суставов, стоп с целью диагностики и оценки поражения опорно-двигательного аппарата (при наличии показаний);

- УЗИ органов брюшной полости и почек;

- консультации офтальмолога, невролога, ортопеда и других специалистов при наличии показаний.

Следование обоснованному алгоритму обследования позволит своевременно выявлять осложнения синдрома Коккейна и проводить возможную медицинскую коррекцию, максимально сохраняя качество жизни пациентов.

## Дополнительная информация

Источники финансирования. Работа выполнена по инициативе авторов без привлечения финансирования.

Конфликт интересов. Авторы декларируют отсутствие явных и потенциальных конфликтов интересов, связанных с содержанием настоящей статьи.

Участие авторов. Все авторы одобрили финальную версию статьи перед публикацией, выразили согласие нести ответственность за все аспекты работы, подразумевающую надлежащие изучение и решение вопросов, связанных с точностью или добросовестностью любой части работы.

Согласие пациента. Законные представители пациентов добровольно подписали согласие на публикацию фотографий и персональной медицинской информации в обезличенной форме на участие в исследовании (одобрено локальным этическим комитетом ФГАУО ВО Первый Московский государственный медицинский университет им. И.М. Сеченова Минздрава России (Сеченовский Университет); выписка из протокола №06-23 от 06.04.2023 г.).
